# Association of Fast-Food Intake with Depressive and Anxiety Symptoms among Young Adults: A Pilot Study

**DOI:** 10.3390/nu16193317

**Published:** 2024-09-30

**Authors:** Wai-Kin Tang, Jetty Chung-Yung Lee

**Affiliations:** 1 HKU School of Professional and Continuing Education, Hong Kong SAR, China; 20098695@learner.hkuspace.hku.hk; 2 School of Biological Sciences, The University of Hong Kong, Hong Kong SAR, China

**Keywords:** fast food, depression, anxiety, mental health

## Abstract

**Background:** High intake of fast food has been linked to increased risks of both depressive and anxiety disorders. However, associations between individual fast-food items and depressive/anxiety disorders are rarely examined. **Method:** Using cross-sectional survey the association between common fast-food items and depressive/anxiety symptoms among 142 young Hong Kong adults aged 18–27 years old was examined. A qualitative food frequency questionnaire was employed to measure the intake frequency of 22 common fast-food items found in Hong Kong. Occurrence of significant depressive and anxiety symptoms was measured by the Patient Health Questionnaire-9 (PHQ-9) and Generalized Anxiety Disorder-7 (GAD-7), respectively. Primary measures were multivariate-adjusted odds ratios for occurrence of depressive and anxiety symptoms compared with the low intake frequency group for common fast-food items. **Results:** Our observations suggest that frequent intake of high-fat, -sugar, and -sodium fast-foods increased depressive symptoms, while frequent high-fat fast-food intake was associated with anxiety symptoms. However, frequent intake of sugar-free beverages reduced the risk of depressive symptoms. **Conclusions:** Habitual intake of certain fast foods were related to depressive/anxiety symptoms in young adults.

## 1. Introduction

Depressive and anxiety disorders are among the most prevalent and disabling mental health conditions that are on the rise globally [1,2]. A recent study found that over half of university students in Hong Kong experienced depressive and anxiety symptoms to some extent [3], which is higher than the global prevalence of 34% and 31% for depressive and anxiety symptoms, respectively [4]. Depressive and anxiety disorders exhibit high co-morbidity and contribute high burdens at both individual and societal levels [5]. They can influence various aspects of an individual’s daily performance, including the ability of fulfilling responsibilities in family and work settings and in the society [6]. They impair quality of life and incur economic burden on the patient’s family and society due to increased medical expenses and decreased productivity [7]. Onset and progression of depression and anxiety disorders are the results of a complex interaction between genetic, psychological, biological, and environmental factors [8]. Previous studies have shown that gender [9], body mass index [10], physical activity level [11,12], sleep duration [13] and quality [14], screen time [15,16], household income [17], alcohol intake [18], and tobacco use [19] are associated with the occurrence of depressive/anxiety symptoms. A growing body of research found that there is a relationship between diet and depressive/anxiety disorders. One meta-analysis of sixteen randomized controlled trials revealed significant reduction of subclinical depressive symptoms through dietary intervention [20] by restricting high-fat and high-sugar food intake and replacing them with high-fiber and nutrient-dense food [20]. Such intervention also reduced bodyweight and improved nutritional intake. Moreover, an association between healthy eating patterns, which consist of higher intake of vegetables, fruit, whole grains, fish, legumes, and unprocessed meat, and decreased subclinical anxiety symptoms has been identified by a recent scoping review [21]. Emerging studies have shown positive correlations between consumption of fast food and occurrence of depressive/anxiety symptoms [22,23,24,25].

Hong Kong is a cosmopolitan city with long working hours. It is a dense city (6910 people per square kilometer) where living space is small with a poverty rate of 20%. More so, one adult in four suffers from depression and anxiety. Under these conditions and financial burden, many are food insecure and opt for satiety over nutrition when choosing food [26,27,28]. The relationship between fast-food intake and occurrence of depressive/anxiety symptoms is warranted in young adults in Hong Kong given the high popularity and heightened prevalence of depressive and anxiety symptoms among them. However, the current studies mainly concentrated on examining the relationship between depressive/anxiety symptoms and overall fast-food consumption as a food group, whereas there is a lack of investigation into the individual food items within the fast-food category. In addition, despite the high comorbidity between depressive and anxiety disorders, most of the previous studies have only focused on the effect of fast food on depression. It is hypothesized that consumption frequencies of different fast-food items and categories have varying relationships with the occurrence of depressive and anxiety symptoms. The aim of the present study is to investigate the association between the consumption frequency of common fast-food items and categories with the occurrence of depressive and anxiety symptoms among Hong Kong young adults.

## 2. Materials and Methods

### 2.1. Study Participants

This cross-sectional study was conducted between February and April 2024 in Hong Kong. The sample size required was statistically estimated by G*Power software version 3.1.9.7 (Universität Düsseldorf, Germany), where 102 responses will allow an error of 5%. One hundred and forty-two university students studying in Hong Kong were recruited using the convenience sampling method via social media and online forums to participate the study. Participants completed an online questionnaire through the Google Form platform. The survey was voluntary and anonymous. All data were self-reported. The study’s inclusion criteria were university students who are studying in Hong Kong and above 18 years of age. Participants under 18 years of age, those with prior diagnoses of mental disorders, and those who did not provide informed consent were excluded from the study. This study was approved by the University of Hong Kong School of Professional and Continuing Education Research Ethics Committee. All participants were informed about the study objectives and provided with informed consent prior to the data collection process.

### 2.2. Assessment of Depressive Symptoms

Depressive symptoms were determined by the nine-item Patient Health Questionnaire (PHQ-9, American Psychological Association) [29]. It is a validated self-administered screening tool designed to measure the occurrence of depressive symptoms and includes rating statements like, for example, “Little interest or pleasure in doing things” and “Poor appetite or overeating” in the past two weeks (rated on a four-point Likert scale response format: “0 = Never” to “3 = Nearly every day”). The PHQ-9 score for each respondent was obtained by summing their response scores to all nine items, with a total possible score range of zero to 27. A PHQ-9 score ≥ 10 indicates moderate or above normal level of depressive symptoms and is considered as having significant depressive symptoms in this study. In this study, Cronbach’s alpha reliability coefficient was 0.91 for the PHQ-9.

### 2.3. Assessment of Anxiety Symptoms

Anxiety symptoms were determined by the seven-item Generalized Anxiety Disorder-7 (GAD-7, Anxiety and Depression Association of America) [30]. It is a validated self-administered screening tool designed to measure the occurrence of anxiety symptoms, with rating statements like, for example “Feeling nervous, anxious or on edge” and “Trouble relaxing” in the past two weeks (rated on a four-point Likert scale response format: “0 = Never” to “3 = Nearly every day”). The GAD-7 score for each respondent was obtained by summing their response scores to all seven items, with a total possible score range of zero to 21. A GAD-7 score ≥ 10 indicates moderate or above average level of anxiety symptoms and is considered as having significant anxiety symptoms in this study. In this study, Cronbach’s alpha reliability coefficient was 0.89 for the GAD-7.

### 2.4. Dietary Assessment

Participants were surveyed using a qualitative food frequency questionnaire (Supplementary Material S1) to assess their past intake frequency of 22 common fast-food items over a period of three months. These fast-food items are popular in the Hong Kong population such as beef burgers, fish burgers, fries, fried chicken (including nuggets), hot dogs, pizza, sandwiches, spaghetti, wraps, carbonated beverages (regular), carbonated beverages (sugar-free), coffee, hot chocolate, hot/iced lemon tea (with sugar), hot/iced lemon tea (sugar-free), milk shakes, milk tea, tea, Boba (bubble) tea, cake, ice cream, and ready-made dessert pie. Participants were asked about the usual consumption frequency of each fast-food item in the last three months, which is rated on a five-point scale: “Never”, “1–4 times a month”, “Several times a week”, “Daily”, and “Several times a day”. In the present study, Cronbach’s alpha reliability coefficient was 0.68 for the food frequency questionnaire.

### 2.5. Covariates

Gender, age, body mass index (BMI), household income, physical activity, sleep time, sleep quality, screen time, alcohol intake, and tobacco use were considered as covariates in this study. BMI was calculated by the formula weight in kg/(height in m)^2^ from participants’ self-reported weight and height. Household income was defined as the average monthly household income in Hong Kong dollars. Physical activity was measured by the number of days the respondent participated in physical activities lasting 30 min or longer, which were sufficient to increase their breathing rate, in the last seven days. Sleep time was defined as average duration of sleep per day. Sleep quality was assessed using a single-item subjective sleep quality scale. Participants were asked to rate their sleep quality on an 11-point Likert scale, from ‘”0 = terrible” to “10 = excellent” [31]. Screen time was defined as average daily time spent on devices with display screens, such as a smart phones. Alcohol consumption was assessed qualitatively on a five-point scale: “Never”, “1–4 times a month”, “Several times a week”, “Daily”, and “Several times a day”. Tobacco use was defined as a current user of tobacco products.

### 2.6. Statistical Analysis

Data analysis was conducted using SPSS 26 (IBM Corp., Armonk, NY, USA). All tests performed were two-sided. Statistical significance was set at *p* < 0.05.

Descriptive characteristics were expressed as percentage for categorical variables and mean ± standard deviation (SD) for continuous variables. Categorical variables were compared using the chi-square test. Continuous variables were tested for normality by the Shapiro–Wilk test. The independent *t*-test was used to compare parametric continuous variables between groups, and the Mann–Whitney U test was used to compare non-parametric continuous variables between groups.

Logistic regression analyses were performed to test the association between occurrence of depressive/anxiety symptoms and the frequency level of each of the 22 fast food items. The intake frequency level of each fast-food item was categorized into three groups based on the frequency of their usual consumption in the last three months: “Never = Low”, “1–4 times a month = Moderate”, “Several times a week or more = High”. As the occurrence of depressive and anxiety symptoms is known to be influenced by socio-economic status and lifestyle factors, three statistical models were used in the analysis. **Model 1** is the crude model with no adjustment for covariates. **Model 2** is a multivariate model with adjustment for age and gender. **Model 3** is based on **Model 2** with further adjustments for BMI, household income, physical activity level, sleep duration, sleep quality, screen time, alcohol consumption, and tobacco use. Odds ratios (ORs) and their 95% confidence intervals (95% CIs) were calculated using the low intake group as a reference.

Logistic regression analyses were also performed to test the association between occurrence of depressive/anxiety symptoms and high-fat, high-sugar, or high-sodium fast foods, as categorized in Table 1. The intake levels of each category were repeated according to the frequency test of individual fast foods with the three statistical models described above. The nutritional compositions of fast-food items were extracted from the Nutrient Information Inquiry System by the Hong Kong Centre for Food Safety (HKCFS) [32], and FoodData Central by the United States Department of Agriculture (USDA) [33]. The fast-food items were categorized into high fat (more than or equal to 20 g per 100 g of food or more than or equal to 20 g per 100 mL of beverage), high sugar (more than or equal to 15 g per 100 g of food or more than or equal to 7.5 g per 100 mL of beverage), and high sodium (more than or equal to 600 mg per 100 g of food or more than or equal to 300 mg per 100 mL of beverage) food, according to the guideline published by the Hong Kong Centre for Food Safety, Hong Kong Government. Based on a 2000 Kcal daily diet intake, the upper limit should not exceed 60 g of fat plus 20 g saturated fat and 2.2 g trans-fat, 50 g for sugar, and 300 mg of sodium [34].

## 3. Results

### 3.1. Characteristics of the Participants

Table 2 presents the demographic characteristics of participants. Among the 142 participants, 37.3% were male, and the mean age was 20.0 ± 1.7 years old. Compared with the participants without significant depressive symptoms, those with significant depressive symptoms were likely to be less physically active, have less sleep time, have lower sleep quality, have higher screen time, have higher alcohol consumption, and be tobacco users. For anxiety symptoms, those with significant anxiety symptoms were likely to have higher BMI, be less physically active, have lower sleep quality, and have higher screen time when compared with those without significant anxiety symptoms.

### 3.2. Fast Food Items Categorization

The total fat, sugar, and sodium contents of fast-food items were extracted from food nutrition databases by HKCFS and USDA and presented in Table 3. The total fat, sugar, and sodium contents of the 22 food items were compared with high-fat, high-sugar, and high-sodium food definitions and nutrient calculator provided by the HKCFS [34] (Table 1). Accordingly, beef burgers and fried chicken were categorized into the high-fat fast-food category; regular carbonated beverages, hot/iced lemon tea (with sugar), milk shakes, bubble tea, cake, ice cream, and ready-made dessert pie were categorized into the high-sugar fast food category; fish burgers, fried chicken, and hot dogs were categorized into the high-sodium fast food category. Among the fast-food items that exceed fat of 20 g per 100 g were fried chicken (28.00 g per 100 g) and beef burger (22.10 g per 100 g). Fried chicken had the highest sodium level (1040 mg per 100 g), while the sodium level of beef burger was 327 mg per 100 g, which was below the limit (Table 1). However, fish burger contained 602 mg sodium per 100 g and hot dog contained 684 mg per 100 g, which exceeded the limit of 600 mg per 100 g. As for food with sugar levels that exceeded the limit, they included dessert pie (21.40 g per 100 g), ice cream (21.22 per 100 g), hot/ice lemon tea with sugar (8.00 g per 100 g), bubble tea (9.03 g per 100 mL), and milk shake (17.80 g per 100 mL). As described below, these food and beverage items were highly related to the odds of depressive and anxiety symptoms.

### 3.3. Fast Food Item Intake and Depressive Symptoms

The ORs of fast-food items intake with occurrence of depressive symptoms are listed in Table 4. Based on Model 3, high intake frequency of beef burgers (OR: 10.82; 95% CI: 2.46, 47.60), moderate intake frequency of fish burgers (OR: 7.37; 95% CI: 1.15, 47.46), moderate (OR: 7.50; 95% CI: 1.52, 37.12) and high (OR: 39.92; 95% CI: 6.95, 229.45) intake frequency of fries, high intake frequency of fried chicken (OR: 28.35; 95% CI: 3.97, 202.40), high intake frequency of sugar-sweetened carbonated beverages (OR: 10.10; 95% CI: 2.68, 38.05), and high intake frequency of bubble tea (OR: 10.08, 95% CI: 2.35, 43.16) were significantly associated with higher risk of depressive symptoms. In contrast, moderate (OR: 0.04; 95% CI: 0.01, 0.23) and high (OR: 0.02, 95% CI: 0.002, 0.20) intake frequency of salad, high intake frequency of sugar-free carbonated beverages (OR: 0.20; 95% CI: 0.06, 0.72), and moderate intake frequency of tea (OR: 0.18; 95% CI: 0.04, 0.88) were associated with lower risk of depressive symptoms.

### 3.4. Fast Food Item Intake and Anxiety Symptoms

Table 5 presents the ORs of fast-food item intake in relation to the occurrence of anxiety symptoms. Based on Model 3, high intake frequency of beef burgers (OR: 3.73; 95% CI: 1.11, 12.58), fries (OR: 14.71; 95% CI: 1.52, 142.28), fried chicken (OR: 14.71; 95% CI: 1.52, 142.28), milk shakes (OR: 58.28; 95% CI: 3.08, 1104.06), bubble tea (OR: 14.58 95% CI: 2.50, 85.12), ice cream (OR: 6.32; 95% CI: 1.37, 29.23), and pie (OR: 32.75; 95% CI: 1.82, 588.53) were significantly associated with higher risk of having anxiety symptoms.

### 3.5. Fast Food Categories Intake and Depressive Symptoms

The ORs of fast-food categories intake with occurrence of depressive symptoms are listed in Table 6. Based on Model 3, high intake frequency of high-fat fast food (OR: 32.40; 95% CI: 5.23, 200.97) and high-sugar fast food (OR: 13.58, 95% CI: 2.80, 65.99) and moderate (OR: 6.23, 95% CI: 1.08, 38.54) and high (OR: 16.80, 95% CI: 2.58, 109.09) intake frequency of high-sodium fast food were significantly associated with higher risk of having depressive symptoms.

### 3.6. Fast Food Categories Intake and Anxiety Symptoms

The ORs of fast-food categories intake with occurrence of anxiety symptoms are shown in Table 7. Based on Model 3, high intake frequency of high-fat fast food (OR: 6.14; 95% CI: 1.16, 32.46) was significantly associated with higher risk of having depressive symptoms.

## 4. Discussion

This cross-sectional study investigated the association between the occurrence of depressive/anxiety symptoms and the consumption frequency of common fast-food items among young adults in Hong Kong. Our results provided evidence that frequent consumption of high-fat, high-sugar, and high-sodium fast-food items, in particular beef burgers, fries, fried chicken, and bubble tea, were significantly associated with increased risk of developing depressive/anxiety symptoms. In addition, we observed that frequent intake of sugar-free beverages, notably tea, was associated with a lower risk of depressive symptoms.

While it is challenging to make direct comparisons with other studies as the topic is specific, a recent longitudinal study revealed that high intake of fried food is a significant risk factor for depressive and anxiety symptoms in male college students in China [35]. Another study found that enhanced consumption of fried food, particularly fried potatoes, increased the risk of depressive and anxiety symptoms by 12% and 7%, respectively. The group also identified that chronic exposure to acrylamide, a toxic compound formed in frying process, is a contributing factor to the increased risk of mental health outcomes [36]. Moreover, a prospective study revealed that high-fat, high-sugar, and low-fiber diet was significantly related to depressive and anxiety symptoms [37]. These findings coincide with our observation, where consumption of high-fat and fried fast-food items, such as beef burgers, fries, and fried chicken, were associated with a higher risk of depressive and anxiety symptoms.

It is evident that high sugar consumption was related to depressive/anxiety symptoms. A recent meta-analysis of observational studies found significant association between the consumption of sugar-sweetened beverages and a higher risk of depression [38]. A cross-sectional study among Chinese adolescents also showed positive association of sugar-sweetened beverages, sweet desserts, fried food, and fast-food intake with increased risks of depression and anxiety [39]. A similar relationship was also observed in this study particularly, where frequent intake of high-fat fast-food and sugar-sweetened beverages increased the risk of anxiety symptoms.

In contrast, our observations revealed that frequent intake of sugar-free beverages including moderate tea intake reduced the risk of depressive symptoms. This is in line with previous studies, which reported a protective effect of tea on depression. However, it should be noted that our study did not evaluate tea with or without sugar. Nevertheless, in local customs, tea is generally related to Chinese tea without sugar. Furthermore, frequent tea-drinking was found to be associated with reduced risk of depressive symptoms among older Chinese adults [40], while in a cross-sectional Mediterranean islands study, it was found that daily tea intake reduced the risk of depression in elderly people from the Mediterranean islands [41].

Potential underlying mechanisms related to consumption of fast-food items and depressive/anxiety disorders are complex and multifactorial. Fast-food diet has been linked to increased intake of energy, saturated fat, and sugar, as well as reduced intake of omega-3 fatty acids, dietary fiber, vitamin A, vitamin C, and vitamin D [42,43]. These nutritional imbalances are known factors associated with an increased risk of developing depression and anxiety [44,45]. High contents of fat and sugar in certain fast-food items are also a potential contributing factor of the observed association. More so, over-consumption of fat and simple sugars can induce oxidative stress and gut barrier dysfunction and increase production of pro-inflammatory cytokines, such as interleukin-1 (IL-1), IL-6, and tumor necrosis factor-alpha (TNF-α), which are related to neuroinflammation [46,47]. It has been reported in multiple studies that neuroinflammation can negatively affect neurotransmitter (such as dopamine and serotonin) biosynthesis, neuroendocrine function, neurogenesis, and neurocircuitry, which are linked to anxiety and depressive disorders [48,49]. Emerging studies have suggested that vitamin D, known for its antioxidant and anti-inflammatory properties, could play a protective role against depression and anxiety disorders [50]. It is therefore possible that frequent intake of fast-food items that lack vitamin D could contribute to increased risk of depressive and anxiety symptoms. In addition, there have been reports indicating that certain phytochemicals found abundantly in tea exhibit inhibitory effects on neuroinflammation [51,52].

Despite not being assessed in this study, we should note that consumption of fast food may be a “comfort” to some individuals. It has been found that high-fat and high-sugar foods were related to emotional eating [53,54], while fast food (hot dogs, hamburgers, cheeseburgers, fried chicken, pizza) consumption was related to behavioral problems such as anxiety, dizziness, and worthlessness in Norwegian adolescents [55]. Additionally, salty snacks, e.g., chips and popcorns, were significantly related to higher risk of self-reported psychiatric distress in Iranian children and adolescents [56].

Although this study displayed some potential implications for dietary intervention among people with depressive/anxiety disorders, as it is a small study, several limitations should also be considered while interpreting our findings. Due to the cross-sectional nature of the study, a causal relationship between fast-food items intake and depressive/anxiety symptoms cannot be established. The definition of fast food is broad, and depending on the culture, the categorization is different. Fast food in Hong Kong is a westernized concept that includes food categories listed in Table 3. Besides, we acknowledge that fast-food related dietary data can be prone to bias evaluation. Furthermore, the relationship between fast food items intake and depressive/anxiety symptoms may be bidirectional, as mental health status could affect dietary choices [57]. Also, in this study we were unable to identify confounding factors for those who had low intake of certain fast-food items (beef burgers, fries, fried chicken, sugar-sweetened carbonated beverages, and bubble tea) or consumption of sugar-free beverages to have none or lesser symptoms of depressive and anxiety. Further longitudinal investigations are warranted to understand the mechanisms and clarify the causality of the observed associations. The current study also relied on self-reported and retrospective data that are prone to recall bias. The sampling method employed was not randomized, which may introduce selection bias. In addition, the sample size was small, and it limited the statistical power of the results. Finally, the food frequency questionnaire used was qualitative and did not collect data on portions of fast-food items consumed.

## 5. Conclusions

Our results showed that frequent intake of high-fat and/or high-sugar Hong Kong fast-food diet increased the risk of depressive and/or anxiety symptoms in young adults. In contrast, frequent intake of sugar-free beverages such as tea decreased the risk of depressive symptoms. Regardless, fast-food diet may be categorically different depending on the culture. In-depth longitudinal studies are recommended to confirm the relationship between individual fast food item intake, as suggested in this study, and the occurrence of depressive/anxiety symptoms. Overall, this investigation displayed potential benefits in restricting fast food dietary habits to alleviate and/or prevent the development of depression and anxiety in young adults.

## Figures and Tables

**Table 1:** High-fat, high-sugar, and high-sodium food definitions according to the guideline published by the Hong Kong Centre for Food Safety [34].

	Per 100 g of Food	Per 100 mL of Beverage
Total fat content	≥20 g	≥20 g
Sugar content	≥15 g	≥7.5 g
Sodium content	≥600 mg	≥300 mg

**Table 2:** Demographic characteristics of study participants (*n* = 142).

	All	With Depressive Symptoms	Without Depressive Symptoms	*p* Value ^a^	With Anxiety Symptoms	Without Anxiety Symptoms	*p* Value ^a^
Gender (%)				0.94			0.44
Male	37.30	37.70	37.00		43.30	35.70	
Female	62.70	62.30	63.00		56.70	64.30	
Age (years)	20.00 ± 1.70	20.00 ± 2.50	20.10 ± 1.50	0.21	20.10 ± 1.90	20.10 ± 1.70	0.98
Body mass index (kg/m^2^)	21.40 ± 2.30	21.80 ± 2.70	21.00 ± 1.90	0.22	22.40 ± 3.30	21.10 ± 1.90	0.007 **
Physically active day (days)	1.40 ± 1.60	0.90 ± 1.40	1.70 ± 1.60	<0.001 **	0.60 ± 0.90	1.50 ± 1.60	0.002 **
Sleep duration (hours)	6.50 ± 1.00	6.20 ± 1.10	6.70 ± 0.80	0.001 **	6.20 ± 1.30	6.50 ± 0.90	0.18
Sleep quality score	6.40 ± 2.20	5.20 ± 2.40	7.40 ± 1.60	<0.001 **	5.30 ± 2.40	6.80 ± 2.10	0.002 **
Screen time (hours)	6.40 ± 1.40	6.90 ± 1.30	6.00 ± 1.40	<0.001 **	7.00 ± 1.40	6.20 ± 1.40	0.011 **
Household income (HK$) (%)				0.41			0.79
$15,000–19,999	5.60	9.80	2.50		10.00	4.50	
$20,000–24,999	9.20	8.20	9.90		6.70	9.80	
$25,000–29,999	18.30	18.00	18.50		23.30	17.00	
$30,000–39,999	24.60	24.60	24.70		20.00	25.90	
$40,000–59,999	21.80	18.00	24.70		16.70	23.20	
$60,000–79,999	12.70	16.40	9.90		16.70	11.60	
$80,000–99,999	6.30	4.90	7.40		6.70	6.30	
>$100,000	1.40	0.00	2.50		0.00	1.80	
Alcohol consumption (%)				0.002 **			0.22
Never	40.80	34.40	45.70		33.30	42.90	
Less than once a week	23.90	14.80	30.90		16.70	25.90	
1 to 3 times a week	27.50	34.40	22.20		33.30	25.90	
4 to 6 times a week	6.30	13.10	1.20		13.30	4.50	
Once or more per day	1.40	3.30	0.00		3.30	0.90	
Tobacco use (%)				0.001 **			0.23
Yes	19.00	31.10	9.90		26.70	17.00	
No	81.00	68.90	90.10		73.30	83.00	

Values are mean ± SD. ^a^ Chi-square test for categorical variables, independent *t*-test for normal distribution continuous variables, or Mann–Whitney U test for non-normal continuous variables. ** *p* < 0.01.

**Table 3:** Fat, sugar, and sodium contents of fast-food items calculated from the Food Nutrient Calculator (Centre for Food Safety, Hong Kong SAR Government).

Fast Food Item	Fat Content (g/100 g for Solid Food or g/100 mL for Liquid)	Sugar Content (g/100 g for Solid Food or g/100 mL for Liquid)	Sodium Content (mg/100 g for Solid Food or mg/100 mL for Liquid)
Beef burger	22.10	0.00	327
Fish burger	12.40	3.53	602
Fries	14.00	0.28	280
Fried chicken	28.00	0.00	1040
Hot dogs	14.84	0.00	684
Pizza	9.51	4.02	535
Sandwiches	9.60	2.80	380
Spaghetti	5.50	2.00	410
Wraps	2.42	3.19	348
Carbonated beverages, regular	0.00	11.00	13
Carbonated beverages, sugar-free	0.00	0.00	17
Coffee	2.30	2.00	32
Hot chocolate	1.90	5.60	36
Hot/iced lemon tea, with sugar	0.30	8.00	0
Hot/iced lemon tea, sugar-free	Trace	0.80	0
Milk shake	3.03	17.80	95
Milk tea	2.60	2.80	38
Tea	0.00	0.00	3
Boba (Bubble) tea	0.97	9.03	27
Cake	17.00	16.5	234
Ice cream	11.00	21.22	80
Dessert pie	16.10	21.40	333

**Table 4:** ORs and 95% CIs of having depressive symptoms according to intake frequency of fast-food items.

	Frequency of Intake		
	Low	Moderate	High
Beef burger			
Case, *n* (%)	79 (55.70)	28 (19.70)	35 (24.60)
Model 1	ref	1.23 (0.48–3.12)	25.41 (5.35–45.18) **
Model 2	ref	1.18 (0.45–3.09)	17.26 (5.71–52.17) **
Model 3	ref	1.32 (0.38–4.56)	10.82 (2.46–47.60) **
Fish burger			
Case, *n* (%)	123 (86.60)	14 (9.90)	5 (3.50)
Model 1	ref	6.13 (1.63–23.16) **	6.70 (0.73–61.74)
Model 2	ref	6.26 (1.64–23.93) **	6.93 (0.74–64.60)
Model 3	ref	7.37 (1.15–47.46) *	2.71 (0.17–44.53)
Fries			
Case, *n* (%)	41 (28.90)	45 (31.70)	56 (39.40)
Model 1	ref	5.15 (1.35–19.66) *	51.82 (13.47–199.42) **
Model 2	ref	5.13 (1.32–19.86) *	52.07 (13.47–201.32) **
Model 3	ref	7.50 (1.52–37.12) *	39.92 (6.95–229.45) **
Fried chicken (including nuggets)			
Case, *n* (%)	33 (23.30)	55 (38.70)	54 (38.00)
Model 1	ref	1.85 (0.64–5.32)	11.70 (4.03–33.99) **
Model 2	ref	1.90 (0.65–5.53)	14.91 (4.81–46.27) **
Model 3	ref	3.71 (0.57–24.04)	28.35 (3.97–202.40) **
Hot dogs			
Case, *n* (%)	115 (81.00)	21 (14.80)	6 (4.20)
Model 1	ref	2.00 (0.78–5.13)	1.50 (0.29–7.76)
Model 2	ref	1.97 (0.77–5.08)	1.53 (0.29–7.97)
Model 3	ref	1.45 (0.16–12.96)	1.90 (0.17–21.77)
Pizza			
Case, *n* (%)	99 (69.70)	39 (27.50)	4 (2.80)
Model 1	ref	2.51 (1.18–5.37) *	1.75 (0.24–12.96)
Model 2	ref	2.52 (1.17–5.42) *	1.72 (0.22–13.62)
Model 3	ref	1.94 (0.68–5.56)	0.37 (0.03–4.94)
Sandwiches			
Case, *n* (%)	23 (16.20)	38 (26.80)	81 (57.00)
Model 1	ref	1.33 (0.44–4.03)	2.23 (0.83–6.00)
Model 2	ref	1.34 (0.44–4.06)	2.23 (0.83–6.02)
Model 3	ref	0.65 (0.14–3.10)	1.18 (0.30–4.73)
Spaghetti			
Case, *n* (%)	121 (85.20)	16 (11.30)	5 (3.50)
Model 1	ref	1.77 (0.62–5.05)	0.34 (0.04–3.16)
Model 2	ref	1.75 (0.61–5.03)	0.33 (0.04–3.06)
Model 3	ref	4.29 (0.90–20.55)	0.13 (0.01–1.96)
Wraps			
Case, *n* (%)	60 (42.30)	74 (52.10)	8 (5.60)
Model 1	ref	0.93 (0.47–1.85)	0.41 (0.08–2.18)
Model 2	ref	0.94 (0.46–1.91)	0.41 (0.08–2.24)
Model 3	ref	1.46 (0.53–4.06)	1.18 (0.14–9.97)
Carbonated beverages, regular			
Case, *n* (%)	76 (53.50)	18 (12.70)	48 (33.80)
Model 1	ref	2.21 (0.74–6.57)	11.67 (4.93–27.66) **
Model 2	ref	3.05 (0.96–9.65)	18.56 (6.70–51.38) **
Model 3	ref	3.54 (0.74–16.94)	10.10 (2.68–38.05) **
Carbonated beverages, sugar-free			
Case, *n* (%)	81 (57.00)	24 (16.90)	37 (26.10)
Model 1	ref	0.42 (0.16–1.09)	0.27 (0.11–0.65) **
Model 2	ref	0.37 (0.14–0.99) *	0.25 (0.10–0.60) **
Model 3	ref	0.71 (0.17–2.90)	0.20 (0.06–0.72) *
Coffee			
Case, *n* (%)	61 (43.00)	26 (18.30)	55 (38.70)
Model 1	ref	0.71 (0.81–1.79)	0.51 (0.24–1.08)
Model 2	ref	0.70 (0.27–1.78)	0.51 (0.24–1.09)
Model 3	ref	0.53 (0.15–1.91)	0.47 (0.16–1.35)
Hot chocolate			
Case, *n* (%)	127 (89.40)	11 (7.80)	4 (2.80)
Model 1	ref	1.09 (0.32–3.76)	0.44 (0.04–4.31)
Model 2	ref	1.10 (0.32–3.82)	0.42 (0.04–4.20)
Model 3	ref	1.32 (0.29–6.07)	0.74 (0.03–17.12)
Hot/iced lemon tea, with sugar			
Case, *n* (%)	102 (71.80)	20 (14.10)	20 (14.10)
Model 1	ref	3.00 (1.12–8.03) *	6.00 (2.01–17.89) **
Model 2	ref	3.11 (1.15–8.40) *	6.09 (2.02–18.33) **
Model 3	ref	2.46 (0.62–9.82)	2.65 (0.63–11.12)
Hot/iced lemon tea, sugar-free			
Case, *n* (%)	132 (93.00)	5 (3.50)	5 (3.50)
Model 1	ref	0.88 (0.14–5.43)	0.88 (0.14–5.43)
Model 2	ref	0.89 (0.14–5.53)	0.86 (0.14–5.37)
Model 3	ref	0.51 (0.05–4.98)	0.46 (0.03–6.77)
Milk shake			
Case, *n* (%)	133 (93.70)	6 (4.20)	3 (2.10)
Model 1	ref	0.25 (0.03–2.21)	0.63 (0.06–7.09)
Model 2	ref	0.25 (0.03–2.24)	0.65 (0.06–7.37)
Model 3	ref	0.07 (0.002–1.83)	0.75 (0.02–37.44)
Milk tea			
Case, *n* (%)	114 (80.30)	23 (16.20)	5 (3.50)
Model 1	ref	1.31 (0.53–3.21)	2.14 (0.34–13.30)
Model 2	ref	1.31 (0.53–3.28)	2.10 (0.33–13.33)
Model 3	ref	1.01 (0.31–3.24)	3.59 (0.22–59.13)
Tea			
Case, *n* (%)	112 (78.90)	23 (16.20)	7 (4.90)
Model 1	Ref	0.24 (0.08–0.76) *	2.89 (0.54–15.50)
Model 2	Ref	0.24 (0.08–0.76) *	2.28 (0.51–15.64)
Model 3	Ref	0.18 (0.04–0.88) *	4.16 (0.37–46.40)
Bubble tea			
Case, *n* (%)	57 (40.10)	36 (25.40)	49 (34.50)
Model 1	ref	0.93 (0.36–2.43)	8.63 (3.58–20.78) **
Model 2	ref	1.16 (0.43–3.15)	13.32 (4.79–37.06) **
Model 3	ref	1.94 (0.51–7.44)	10.08 (2.35–43.16) **
Cake			
Case, *n* (%)	137 (96.50)	3 (2.10)	2 (1.40)
Model 1	ref	2.72 (0.24–30.77)	1.36 (0.08–22.23)
Model 2	ref	2.82 (0.25–32.35)	1.38 (0.08–22.87)
Model 3	ref	12.62 (0.15–1043.36)	3.36 (0.09–121.22)
Ice cream			
Case, *n* (%)	108 (76.00)	22 (15.50)	12 (8.50)
Model 1	ref	3.50 (1.35–9.11) **	22.00 (2.73–177.13) **
Model 2	ref	3.72 (1.39–9.91) **	23.03 (2.83–187.12) **
Model 3	ref	2.59 (0.81–8.34)	9.47 (0.92–97.96)
Pie			
Case, *n* (%)	126 (88.70)	11 (7.80)	5 (3.50)
Model 1	ref	1.14 (0.33–3.96)	2.07 (0.33–12.80)
Model 2	ref	1.12 (0.32–3.93)	1.99 (0.32–12.59)
Model 3	ref	1.09 (0.19–6.43)	0.59 (0.04–9.35)

Model 1: crude model. Model 2: multivariate model, adjusted for age and gender. Model 3: Model 2 + BMI, household income, physical activity level, sleep duration, sleep quality, screen time, alcoholic beverages consumption, and tobacco use. Low intake: Never. Moderate intake: 1–4 times a week. High intake: Several times a week or more. * *p* < 0.05, ** *p* < 0.01.

**Table 5:** ORs and 95% CIs of having anxiety symptoms according to intake frequency of fast-food items.

	Frequency of Intake		
	Low	Moderate	High
Beef burger			
Case, *n* (%)	79 (55.70)	28 (19.70)	35 (24.60)
Model 1	ref	1.69 (0.51–5.56)	6.55 (2.51–17.13) **
Model 2	ref	1.62 (0.48–5.46)	6.45 (2.45–17.00) **
Model 3	ref	1.70 (0.43–6.75)	3.73 (1.11–12.58) *
Fish burger			
Case, *n* (%)	123 (86.60)	14 (9.90)	5 (3.50)
Model 1	ref	1.13 (0.29–4.35)	6.19 (0.98–39.11)
Model 2	ref	1.09 (0.28–4.29)	5.87 (0.92–37.51)
Model 3	ref	0.88 (0.17–4.54)	3.41 (0.35–33.01)
Fries			
Case, *n* (%)	41 (28.90)	45 (31.70)	56 (39.40)
Model 1	ref	7.37 (0.87–62.74)	25.88 (3.31–202.16) **
Model 2	ref	7.16 (0.83–61.59)	25.61 (3.27–200.53) **
Model 3	ref	6.32 (0.67–59.70)	14.71 (1.52–142.28) *
Fried chicken (including nuggets)			
Case, *n* (%)	33 (23.30)	55 (38.70)	54 (38.00)
Model 1	ref	5.45 (0.65–45.69)	20.36 (2.59–160.44) **
Model 2	ref	5.67 (0.67–48.19)	29.19 (3.49–244.46) **
Model 3	ref	6.32 (0.67–56.70)	14.71 (1.52–142.28) *
Hot dogs			
Case, *n* (%)	115 (81.00)	21 (14.80)	6 (4.20)
Model 1	ref	1.79 (0.62–5.16)	4.48 (0.84–23.75)
Model 2	ref	1.78 (0.61–5.18)	4.34 (0.81–23.34)
Model 3	ref	0.21 (0.03–1.69)	0.30 (0.03–3.08)
Pizza			
Case, *n* (%)	99 (69.70)	39 (27.50)	4 (2.80)
Model 1	ref	1.45 (0.61–3.49)	1.40 (0.14–14.25)
Model 2	ref	1.49 (0.61–3.63)	1.30 (0.12–14.42)
Model 3	ref	0.93 (0.31–2.75)	0.41 (0.03–6.34)
Sandwiches			
Case, *n* (%)	23 (16.20)	38 (26.80)	81 (57.00)
Model 1	ref	0.89 (0.22–3.56)	1.56 (0.47–5.12)
Model 2	ref	0.94 (0.23–3.80)	1.62 (0.49–5.37)
Model 3	ref	0.65 (0.12–3.53)	1.12 (0.26–4.83)
Spaghetti			
Case, *n* (%)	121 (85.20)	16 (11.30)	5 (3.50)
Model 1	ref	0.50 (0.11–2.33)	0.87 (0.09–8.12)
Model 2	ref	0.47 (0.10–2.22)	0.85 (0.09–8.11)
Model 3	ref	0.33 (0.05–2.22)	0.98 (0.08–12.05)
Wraps			
Case, *n* (%)	60 (42.30)	74 (52.10)	8 (5.60)
Model 1	ref	0.70 (0.31–1.60)	0.43 (0.05–3.77)
Model 2	ref	0.73 (0.31–1.72)	0.45 (0.05–4.04)
Model 3	ref	0.88 (0.31–2.49)	0.56 (0.05–6.29)
Carbonated beverages, regular			
Case, *n* (%)	76 (53.50)	18 (12.70)	48 (33.80)
Model 1	ref	2.82 (0.73–10.93)	6.46 (2.45–17.02) **
Model 2	ref	3.05 (0.77–2.17)	7.09 (2.55–19.47) **
Model 3	ref	2.45 (0.49–12.27)	3.00 (0.82–11.03)
Carbonated beverages, sugar-free			
Case, *n* (%)	81 (57.00)	24 (16.90)	37 (26.10)
Model 1	ref	0.44 (0.12–1.62)	0.71 (0.27–1.87)
Model 2	ref	0.45 (0.12–1.71)	0.75 (0.28–2.03)
Model 3	ref	0.66 (0.11–3.83)	1.42 (0.40–5.04)
Coffee			
Case, *n* (%)	61 (43.00)	26 (18.30)	55 (38.70)
Model 1	ref	0.56 (0.17–1.88)	0.77 (0.32–1.85)
Model 2	ref	0.56 (0.17–1.92)	0.75 (0.31–1.83)
Model 3	ref	0.50 (0.12–2.06)	1.02 (0.35–2.96)
Hot chocolate			
Case, *n* (%)	127 (89.40)	11 (7.80)	4 (2.80)
Model 1	ref	1.53 (0.38–6.19)	4.08 (0.55–30.40)
Model 2	ref	1.45 (0.35–5.93)	3.96 (0.53–29.91)
Model 3	ref	1.80 (0.35–9.35)	13.76 (0.89–214.02)
Hot/iced lemon tea, with sugar			
Case, *n* (%)	102 (71.80)	20 (14.10)	20 (14.10)
Model 1	ref	0.52 (0.11–2.44)	4.67 (1.69–12.86) **
Model 2	ref	0.50 (0.11–2.37)	4.59 (1.67–12.78) **
Model 3	ref	0.14 (0.02–1.05)	0.35 (0.51–6.85)
Hot/iced lemon tea, sugar-free			
Case, *n* (%)	132 (93.00)	5 (3.50)	5 (3.50)
Model 1	ref	0.97 (0.10–9.06)	2.59 (0.41–16.30)
Model 2	ref	1.04 (0.11–9.80)	2.81 (0.44–18.00)
Model 3	ref	0.63 (0.05–7.58)	3.81 (0.33–43.58)
Milk shake			
Case, *n* (%)	133 (93.70)	6 (4.20)	3 (2.10)
Model 1	ref	0.79 (0.09–7.00)	7.85 (0.69–89.85)
Model 2	ref	0.80 (0.09–7.21)	8.20 (0.70–95.70)
Model 3	ref	0.22 (0.01–3.45)	58.28 (3.08–1104.06) **
Milk tea			
Case, *n* (%)	114 (80.30)	23 (16.20)	5 (3.50)
Model 1	ref	1.04 (0.35–3.09)	0.94 (0.10–8.78)
Model 2	ref	0.96 (0.32–2.92)	0.86 (0.09–8.20)
Model 3	ref	0.84 (0.24–2.89)	0.76 (0.07–8.84)
Tea			
Case, *n* (%)	112 (78.90)	23 (16.20)	7 (4.90)
Model 1	ref	0.30 (0.07–1.36)	0.53 (0.06–4.55)
Model 2	ref	0.30 (0.07–1.35)	0.53 (0.06–4.75)
Model 3	ref	0.51 (0.09–2.86)	0.23 (0.02–3.02)
Bubble tea			
Case, *n* (%)	57 (40.10)	36 (25.40)	49 (34.50)
Model 1	ref	1.30 (0.33–5.20)	7.80 (2.65–22.93) **
Model 2	ref	1.89 (0.44–8.03)	17.48 (4.53–67.47) **
Model 3	ref	1.99 (0.39–10.20)	14.58 (2.50–85.12) **
Cake			
Case, *n* (%)	137 (96.50)	3 (2.10)	2 (1.40)
Model 1	ref	1.95 (0.17–22.25)	3.89 (0.24–64.19)
Model 2	ref	2.30 (0.20–27.03)	4.61 (0.27–77.99)
Model 3	ref	2.38 (0.12–46.40)	9.92 (0.30–326.67)
Ice cream			
Case, *n* (%)	108 (76.00)	22 (15.50)	12 (8.50)
Model 1	ref	1.57 (0.51–4.84)	10.71 (2.90–39.57) **
Model 2	ref	1.79 (0.56–5.73)	11.92 (3.11–45.69) **
Model 3	ref	1.21 (0.31–4.72)	6.32 (1.37–29.23) *
Pie			
Case, *n* (%)	126 (88.70)	11 (7.80)	5 (3.50)
Model 1	ref	1.68 (0.41–6.82)	17.91 (1.91–167.83) *
Model 2	ref	1.71 (0.41–7.16)	20.02 (2.05–194.67) *
Model 3	ref	1.85 (0.36–9.54)	32.75 (1.82–588.53) *

Model 1: crude model. Model 2: multivariate model, adjusted for age and gender. Model 3: Model 2 + BMI, household income, physical activity level, sleep duration, sleep quality, screen time, alcoholic beverages consumption, and tobacco use. Low intake: Never. Moderate intake: 1–4 times a week. High intake: Several times a week or more. * *p* < 0.05, ** *p* < 0.01.

**Table 6:** ORs and 95% CIs of having depressive symptoms according to intake frequency of fast-food categories.

	Frequency of Intake		
	Low	Moderate	High
High fat fast food			
Case, *n* (%)	26 (18.30)	49 (34.50)	67 (47.20)
Model 1	ref	1.50 (0.36–6.20)	22.55 (6.01–84.66) **
Model 2	ref	1.48 (0.35–6.18)	23.81 (6.23–91.30) **
Model 3	ref	3.04 (0.50–18.35)	32.40 (5.23–200.97) **
High sugar fast food			
Case, *n* (%)	30 (21.10)	42 (29.60)	70 (49.30)
Model 1	ref	1.77 (0.49–6.41)	14.18 (4.41–45.57) **
Model 2	ref	1.80 (0.49–6.55)	14.52 (4.48–47.04) **
Model 3	ref	3.52 (0.69–17.94)	13.58 (2.80–65.99) **
High sodium fast food			
Case, *n* (%)	26 (18.30)	57 (40.10)	59 (41.60)
Model 1	ref	3.54 (0.94–13.33)	16.14 (4.31–60.50) **
Model 2	ref	3.59 (0.95–13.61)	18.48 (4.77–71.57) **
Model 3	ref	6.23 (1.08–38.54) *	16.80 (2.58–109.09) **

Model 1: crude model. Model 2: multivariate model, adjusted for age and gender. Model 3: Model 2 + BMI, household income, physical activity level, sleep duration, sleep quality, screen time, alcoholic beverages consumption, and tobacco use. Low intake: Never. Moderate intake: 1–4 times a week. High intake: Several times a week or more. * *p* < 0.05, ** *p* < 0.01.

**Table 7:** ORs and 95% CIs of having anxiety symptoms according to intake frequency of fast-food categories.

	Frequency of Intake		
	Low	Moderate	High
High fat food			
Case, *n* (%)	26 (18.30)	49 (34.50)	67 (47.20)
Model 1	ref	2.00 (0.38–10.41)	9.16 (2.00–41.93) **
Model 2	ref	2.00 (0.38–10.45)	9.49 (2.06–43.78) **
Model 3	ref	2.09 (0.36–11.97)	6.14 (1.16–32.46) *
High sugar fast food			
Case, *n* (%)	30 (21.10)	42 (29.60)	70 (49.30)
Model 1	ref	1.50 (0.34–6.54)	6.37 (1.76–22.99) **
Model 2	ref	1.48 (0.34–6.51)	6.34 (1.75–22.97) **
Model 3	ref	1.27 (0.25–6.33)	3.68 (0.83–16.38)
High sodium fast food			
Case, *n* (%)	26 (18.30)	57 (40.10)	59 (41.60)
Model 1	ref	3.55 (0.74–17.04)	7.67 (1.65–35.56) **
Model 2	ref	3.59 (0.74–17.29)	8.39 (1.77–39.72) **
Model 3	ref	3.86 (0.71–21.00)	4.85 (0.88–26.93)

Model 1: crude model. Model 2: multivariate model, adjusted for age and gender. Model 3: Model 2 + BMI, household income, physical activity level, sleep duration, sleep quality, screen time, alcoholic beverages consumption, and tobacco use. Low intake: Never. Moderate intake: 1–4 times a week. High intake: Several times a week or more. * *p* < 0.05, ** *p* < 0.01.

## Data Availability

The data presented in this study are available on request from the corresponding author due to ethical reasons.

## Supplementary Materials

The following supporting information can be downloaded at https://www.mdpi.com/article/10.3390/nu16193317/s1: Questionnaire S1: Questionnaire.
