# Supplementary material for: Association of Fast-Food Intake with Depressive and Anxiety Symptoms among Young Adults: A Pilot Study
Source: Nutrients. 2024 Sep 30;16(19):3317. doi: 10.3390/nu16193317 (PMC11478624; doi:10.3390/nu16193317)
Supplement: Supplementary file 1 [file nutrients-16-03317-s001.zip › nutrients-3160055-supplementary.pdf]

Section A. Basic Information

1.

What is your gender?

Male ☐ Female ☐

2.

What is your age?

____

3.

What is your height in centimeter?

___________cm

4.

What is your weight in kilogram?

___________kg

5.

Are you currently a university student?

Yes ☐ No ☐

6.

You are currently studying

Undergraduate ☐ Postgraduate ☐ Other (Please specify:________) ☐

7.

What is your average monthly household income? (HKD)

< 2,000	☐
2,000 – 3,999	☐
4,000 – 5,999	☐
6,000 – 7,999	☐
8,000 – 9,999	☐
10,000 – 14,999	☐
15,000 – 19,999	☐
20,000 – 24,999	☐
25,000 – 29,999	☐
30,000 – 39,999	☐
40,000 – 59,999	☐
60,000 – 79,999	☐
80,000 – 99,999	☐
≥ 100,000	☐
Don't know/ Prefer not to answer	☐

8.

Have you ever been diagnosed with the following diseases or medical conditions?

	Yes	No
1. Cancer		
2. Diabetes		
3. Hypertension		
4. Hypercholesterolemia or hyperlipidemia		
5. Mental health conditions (such as depression, anxiety, eating disorders, or bipolar disorders)		

Section B. Lifestyle

1.

In the past 7 days, on how many days have you done a total of 30 minutes or more of physical activity, which was enough to raise your breathing rate?

This may include sport, exercise, and brisk walking or cycling for recreation or to get to and from places, but should not include housework or physical activity that may be part of your job.

0	1	2	3	4	5	6	7
☐	☐	☐	☐	☐	☐	☐	☐

2.

How many hours do you sleep per day, on average?

____________Hours

3.

How would you rate your overall sleep quality?

Terrible										Excellent
0	1	2	3	4	5	6	7	8	9	10
☐	☐	☐	☐	☐	☐	☐	☐	☐	☐	☐

4.

What is your average screen time per day?

“Screen time” refers to the total time spent on a device with display screen, such as smartphones, televisions and computers.

____________Hours

5.

How often do you have a drink containing alcohol?

Never	Less than once a week	1 to 3 times a week	4 to 6 times a week	Once or more than once per day
☐	☐	☐	☐	☐

6.

Do you regularly use tobacco products, such as cigarettes, cigars, e-cigarettes and tobacco pipes?

Yes	No
☐	☐

Section C. Dietary Information

“Fast food” refers to meals that are served quickly. It is designed for quick and convenient consumption. In this study, we will focus on fast food that is sold in western fast food chain restaurants, such as McDonald’s and KFC. Some typical example of western fast food are fries, burgers and fried chickens.

In the following section, we will ask about your dietary habits and perception related to western fast food.

1.

In the last 3 months, how often did you buy and eat the food listed below from a western fast food chain

	Never	1 to 4 times a month	Several times a week	Daily	Several times a day
Food					
1. Burger					
2. Fish burger					
3. Fries					
4. Fried chicken (including nuggets)					
5. Hot dogs					
6. Pizza					
7. Sandwiches (including Submarine sandwiches)					
8. Salad					
9. Spaghetti					
10. Wraps					
Beverages					
11. Carbonated beverages, regular					
12. Carbonated beverages, sugar-free					
13. Coffee					
14. Hot chocolate					
15. Hot/ ice lemon tea, with sugar					
16. Hot/ ice lemon tea, sugar-free					
17. Milk					
18. Milk shake					
19. Milk tea					
20. Tea					
21. Bubble tea					
Other					
22. Cake					
23. Ice creams					
24. Pies					
25. Yoghurt					

2.

Thinking about the last 3 months, how often do you engage in the following actions when ordering fast food in a chain western fast food restaurant?

	Never	1 to 4 times a month	Several times a week	Daily	Several times a day
1. Choose healthier food options (e.g. corn cup instead of fries)					
2. Order add-on food (e.g. extra apple pie) along with meal sets					
3. Upgrade fries size					
4. Order extra-large meal sets					

3.
Please indicate your level of agreement with the following statements.

	Strongly Disagree	Disagree	Neutral	Agree	Strongly Agree
1. Western fast food is my primary source of nutrition					
2. I often crave for western fast food					
3. I often feel excessively full after eating western fast food					

4.
Do you prefer western fast food over other food?

Yes ☐ No ☐

5.
If the answer to the above question is "Yes", what is the primary reason for preferring western fast food over other food?

1. Convenience	
2. Eat with friends	
3. Good variety of food	
4. Good service	
5. Good environment	
6. Habit	
7. Price is attractive	
8. Satiety after consumption	
9. Taste good	
10. Other (Please specify: ____________________________)	

Section D. Questionnaire about Depression and Anxiety Symptoms

1.

Over the last 2 weeks, how often have you been bothered by any of the following problems?

	Not at all	Several days	More than half the days	Nearly every day
1. Little interest or pleasure in doing things				
2. Feeling down, depressed, or hopeless				
3. Trouble falling or staying asleep, or sleeping too much				
4. Feeling tired or having little energy				
5. Poor appetite or overeating				
6. Feeling bad about yourself — or that you are a failure or have let yourself or your family down				
7. Trouble concentrating on things, such as reading the newspaper or watching television				
8. Moving or speaking so slowly that other people could have noticed? Or the opposite — being so fidgety or restless that you have been moving around a lot more than usual				
9. Thoughts that you would be better off dead or of hurting yourself in some way				

2.
If you checked off any problems, how difficult have these problems made it for you to do your work, take care of things at home, or get along with other people?

Not difficult at all	Somewhat difficult	Very difficult	Extremely difficult
☐	☐	☐	☐

Developed by Drs. Robert L. Spitzer, Janet B.W. Williams, Kurt Kroenke and colleagues, with an educational grant from Pfizer Inc. No permission required to reproduce, translate, display or distribute.

3.

Over the last 2 weeks, how often have you been bothered by any of the following problems?

	Not at all	Several days	More than half the days	Nearly every day
1. Feeling nervous, anxious or on edge				
2. Not being able to stop or control worrying				
3. Worrying too much about different things				
4. Trouble relaxing				
5. Being so restless that it is hard to sit still				
6. Becoming easily annoyed or irritable				
7. Feeling afraid as if something awful might happen				

Developed by Drs. Robert L. Spitzer, Janet B.W. Williams, Kurt Kroenke and colleagues, with an educational grant from Pfizer Inc. No permission required to reproduce, translate, display or distribute.
